# Topology Optimization Method of Stamping Structures Based on the Directional Density Field

**DOI:** 10.3390/ma17030656

**Published:** 2024-01-29

**Authors:** Zhiling Yuan, Lei Geng, Ningning Wang, Tao Wu, Wei Qi, Yuhua Dai, Jiaqi Huang

**Affiliations:** 1 State Key Laboratory of Engine Reliability, Weifang 261071, China; 2 Weichai Power Co., Ltd., Weifang 261071, China; 3 School of Mechanical Engineering, Shandong University, Jinan 250013, China

**Keywords:** stamping structure, topology optimization, directional density field, truncation threshold

## Abstract

The stamping process produces thin-walled structures that, in general, have uniform wall thickness and no enclosed cavity. However, it is difficult to satisfy the above geometric requirements with the current density-based topology optimization method, since configuring the related geometric constraints is challenging. In order to solve this problem, a topology optimization method for stamping structures based on a directional density field is proposed. Specifically, the directional density field is developed to enable the adding and removing of materials only along the stamping direction, so as to avoid internal voids and concave features. The geometric control for uniform wall thickness is realized by tuning the truncation threshold of the Heaviside projection that processes the directional density field into the 0–1 binary field. At the same time, a calibrated filter radius of the truncation thresholds will facilitate the drawing angle control of the stamping ribs. The effectiveness of the established method has been verified by a number of numerical case studies. Results show that the proposed method can perform topology optimization for stamping structures with tunable uniform thickness and drawing angle control of the ribs. No internal voids or undercuts appear in the results. The results also disclose that a constant truncation threshold increment does not guarantee uniform wall thickness, and varying the threshold increments through surface offset and polynomial fitting is necessary.

## 1. Introduction

Topology optimization, after a few decades of development, has emerged as a multi-disciplinary structural design technology [1], covering the physical aspects of solid mechanics, thermal dynamics, fluid mechanics, magnetics, etc. The topological variable-based interpolations for the above physical governing equations have been developed, as well as the adjoint sensitivity solutions. Accordingly, a variety of specific methods have been proposed for topology optimization that are distinguished by the topological variable definition, including the homogenization method, solid isotropic material with penalization (SIMP) method [1], level set method [2], bidirectional evolutionary structural optimization (BESO) method [3], and some others. Recently, the optimization has expanded from a single-scale structure design to a multi-scale design with lattice geometries [4,5,6]. It has been reported in the literature that incorporating lattices enhances all properties of buckling strength, vibration resistance, convective heat transfer, etc. [7,8,9,10]. Given the practical implementation, topology optimization has been included in the majority of commercial CAD/CAE packages in order to support fast and creative early-stage product development. Increasing the computing scale is a trend that can be used to better execute the algorithms. The real cases, when performing the discretization, normally involve heavy meshes, i.e., at least millions of elements, and topology optimization involves hundreds of iterations and equal numbers of finite element analyses. The total computational effort is oftentimes prohibitively large. High-performance topology optimization running on a supercomputer provides a method capable of optimizing full-scale components with more than 1 billion voxels [11,12]. The optimized full-scale structures have unprecedented structural details and reduce the weight significantly. However, the use of high-performance topology optimization is not exclusive to supercomputers. By exploiting the computing power of GPU, Traff et al. [13] created a simple and efficient GPU-accelerated topology optimization which was able to solve optimization problems with 65.5 million elements in approximately 2 h. Xu et al. [14] accelerated large-scale topology optimization for laser powder bed fusion by configuring PETSc parallel computing, wherein the acceleration effect subject to different amounts of CPU cores was disclosed.

Another aspect that needs highly focused attention is the manufacturability issue. The topological designs are derived by maximizing physical properties subject to a limited amount of material usage. Disordered material distribution, i.e., a complicated geometry, is generally the result; this is due to the extremely large design space. Even to date, manufacturing these complex geometries remains a tough problem. Liu and Ma [15] proposed a feature-based topology optimization algorithm that regularizes the level set front evolution with feature fitting. Some 2.5D feature-based design results were produced that are friendly to 2.5D subtractive machining. Langelaar [16] built an accumulative filter used to ensure accessibility from a specific direction, and the multi-axis scenario was addressed through aggregation, thereby realizing the topology optimization for multi-axis subtractive machining. Gasick and Qian [17] realized the machining-oriented topology optimization in an alternative way: the Helmholtz-type partial differential equation was adopted to derive the inaccessible field, which was eliminated by configuring accessibility constraints. In regard to casting and injection molding, specific topology optimization methods were developed [18,19,20] with the goal of eliminating undercuts or interior voids. For instance, Wang and Kang [21] processed the virtual velocities for level set frontier evolution to retain only the component along the de-molding direction. Regarding additive manufacturing [22,23], its layer-based manufacturing process can better produce complex geometries (including thin-walled structures [24,25] and metamaterials [26,27]), but manufacturability restrictions still exist that should be carefully addressed during the design stage [28,29]. Extensive research efforts were put toward addressing the self-support issue [30,31], material anisotropy issue [32,33,34,35,36,37], connectivity issue of multi-scale structures [38,39], residual stress issue for metal parts [40,41,42,43], etc. These attempts improve the cost-efficiency and manufacturability of the topological parts for additive and hybrid manufacturing [44].

As can be seen from the above, topology optimization methods have been extensively developed for subtractive machining, injection molding/casting, and additive manufacturing. As an alternative manufacturing option, metal forming changes the shape of the workpiece through pressing, during which large stresses and plastic deformation are deeply involved. However, topology optimization for metal forming is less focused than other manufacturing approaches. In stamping, for example, tremendous efforts were put toward optimizing the internal structure of the stamping die [45,46], and the die’s light weight is significant for saving energy and green manufacturing. However, the mechanical performance of the sheet metal workpiece raises more interest for the end user, since the mechanical properties determine the load-bearing capacity during the utilization of the stamped sheet metal. Dienemann et al. [47] proposed a topology optimization method to design stamped parts with uniform thickness. They adopted the smoothing filter to control the minimum thickness and counted on offsetting the median surface to restrict the maximum thickness. However, the median surface is non-derivable in terms of the density variables, and there is no mechanism involved in controlling the maximum drawing angle. The above seems to be the only work that is closely linked to topology optimization for sheet metal stamping, revealing an urgent need for new methods to be proposed. Hence, this paper proposes a new topology optimization method for stamped thin-walled structures and the main contribution lies in the new definition of topological density variables and the effective uniform thickness control and drawing angle control. The directional density field enables directional removal of materials that prevent undercut and interior void features. The constant thickness is realized by setting two truncation thresholds for each column of voxels when processing the directional density field. At the same time, tuning the averaging radius for smoothing the starting truncation thresholds facilitates the maximum drawing angle control. Details of the proposed numerical method will be given in the following sections.

The rest of this article is organized as: Section 2 presents details of the proposed topology optimization method, including the directional density field, the truncation Heaviside projection, the finite element analysis model, the topology optimization problem formulation and its solution, and the offsetting method for uniform thickness control; Section 3 presents the numerical case study in which two cases are investigated to validate the effectiveness of stamping part design while disclosing the effects of uniform thickness control and drawing angle control; Section 4 concludes the article.

## 2. Topology Optimization Method

### 2.1. The Directional Density Field and Definition of the Design Variables

Traditionally for topology optimization, especially the density-based method, the part is discretized with the mesh grids and each of them is assigned a relative density ranging from 0 to 1: 0 means void and 1 represents solid [1]. The relative density is linked to the local material properties and penalization is carried out to prevent intermediate densities. Then, the continuous evolution of the densities carries out the optimization. The free material distribution brings in the extra-large design space, but regarding stamping, the formed parts are cavity-free, thin-walled structures with constant thickness. Imposing the above geometric restrictions with the current density-based topology optimization method is challenging. Hence, in this research, we propose a new definition for the topological densities: the directional density field. For each column of elements along the stamping direction, the relative densities are monotonically and linearly arranged, and two thresholds are designed for each column that truncate the densities to only maintain the median part through Heaviside projections. This median part is assigned as the solid and the thickness can be adjusted by tuning the truncation thresholds.

**Figure 1:**
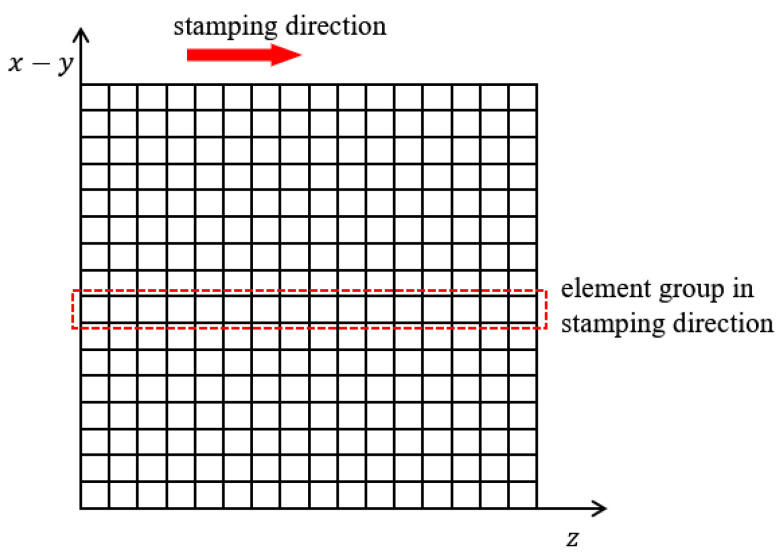
Element grouping along the stamping direction.

**Figure 2:**
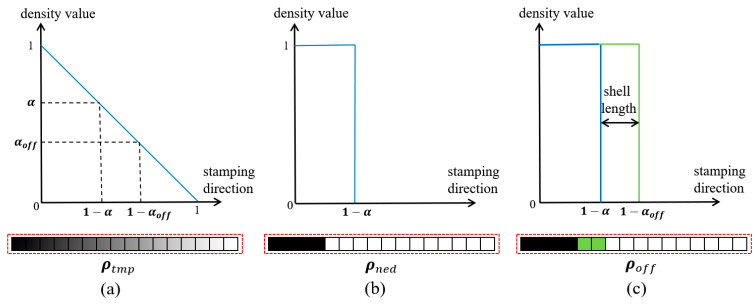
Illustration of the truncation mechanism: graphic description of (**a**) the density vector of $\rho_{tmp}$, (**b**) the density vector of $\rho_{ned}$, and (**c**) the density vector of $\rho_{off}$.

**Figure 3:**
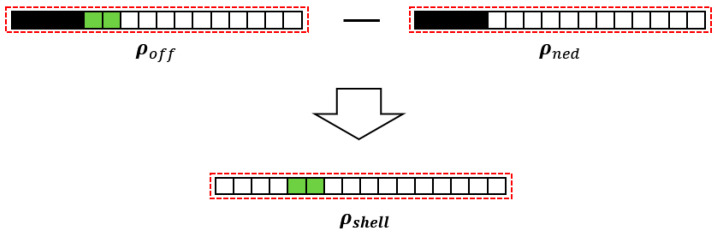
Description of the thickness control.

Specifically, in Figure 1, the discretized domain with nelx×nely×nelz grids has the *z*-axis as the stamping direction. nelx, nely, nelz are short for the number of elements in the *x*-axis, *y*-axis, and *z*-axis direction, respectively. Then, each column of grids along the *z*-axis involves only one design variable and the definition of this design variable is illustrated in Figure 2. The elements along the stamping direction are linearly assigned virtual densities varying from 1 to 0, thus obtaining the column density vector $\rho_{tmp}$, as shown in Figure 2a. Then, a Heaviside projection is performed with the truncation threshold α for binary processing, obtaining the updated density vector: $\rho_{ned}$. Referring to Figure 2b, $\rho_{ned}$ is a binary vector in which the equal-to-1 elements represent solids. Later, the threshold α minus a constant ∆α reaches the second truncation threshold $\alpha_{off}$, and here, performing the Heaviside projection again on $\rho_{tmp}$ with the new truncation threshold derives $\rho_{off}$ as depicted in Figure 2c. $\rho_{off}$ has more equal-to-1 components than $\rho_{ned}$, and the difference is only dependent on ∆α. Finally, by subtracting $\rho_{ned}$ from $\rho_{off}$ through Equation (1), we obtain the physical density vector $\rho_{shell}$ for the stamping part; refer to Figure 3.

(1) ρshell=ρoff−ρned ρoff=tanhβαoff+tanhβρtmp−αofftanhβαoff+tanhβ1−αoff ρned=tanhβα+tanhβρtmp−αtanhβα+tanhβ1−α

In Equation (1), the parameter β determines the projection sharpness, and when β approaches infinity, the step projection effect can be achieved [48]. To avoid the local minimum issue and simultaneously guarantee the differentiability, a small β is normally initiated and its magnitude increases throughout the iterations. In this paper, β has the initial value of 1 and doubles for every 40 iterations until reaching 64. The truncation threshold α controls the solid material distribution and ∆α determines the material thickness along the stamping direction. Therefore, if making ∆α a constant, the formed solid structure will employ a uni-thickness and α would be the only design variable.

### 2.2. The Finite Element Model

Regarding the finite element model, the elemental elastic matrix is calculated through:

(2) $$K_{0}=\int_{-1}^{+1}\int_{-1}^{+1}\int_{-1}^{+1}B^{T}D_{0}Bd\xi_{1}d\xi_{2}d\xi_{3}$$

where $D_{0}$ is the stress–strain relationship, and B represents the strain–displacement matrix. The global elastic matrix can be built through assembling the elemental matrixes:

(3) $$K=\sum_{i=1}^{n}K_{i}({\gamma_{i},\rho }_{i})=\sum_{i=1}^{n}E({\gamma_{i},\rho }_{i})K_{0}$$

where n is the total number of elements. The interpolation term has the following expression [49]:

(4) $$E\left(\gamma_{i},\rho_{i}\right)=\left(E_{min}+\gamma_{i}^{p_{1}}\frac{\rho_{i}}{1+p_{2}\left(1-\rho_{i}\right)}\left(E_{0}-E_{min}\right)\right)$$

in which γ is the design variable distinguishing the solid or void status. ρ means the element density derived by projecting the column truncation thresholds. Note that γ is the topological variable that is consistent for a column of elements and, thus, it eliminates a column of elements from the design when approaching 0. In contrast, ρ is the element-wise density that is affected by the thickness distribution inside the column. $E_{0}$ is Young’s modulus of the based material, and $E_{min}$ is a small positive constant for the void to avoid stiffness singularity. $p_{1}$ and $p_{2}$ are the penalization terms for achieving black and white designs. Finally, the structural displacement solution is derived by solving Equation (5).

(5) KU=F

U and F are the global displacement and force vectors, respectively.

### 2.3. The Optimization Model

This work conducts the stamping structure design based on the compliance-minimization problem and, thus, the optimization problem is formulated as:

(6) $$min\;:\;c\left(\gamma ,\alpha \right)=U^{T}KU\;s.t.\;:\;KU=F\;:\;\frac{V\left(\gamma ,\alpha \right)}{V_{0}}\leq f\;:\;∆\alpha <\alpha \leq 1\;:\;0<\gamma \leq 1$$

where, c means the structural compliance, α is the variable vector of the starting truncation thresholds, $V\left(\gamma ,\alpha \right)$ and $V_{0}$ are the measured solid volume and the design domain volume, respectively. f means the maximum allowable volume fraction.

The sensitivity of the objective function with respect to $\alpha_{i}$ can be calculated through:

(7) $$\frac{\partial c\left(\gamma ,\alpha \right)}{\partial \alpha_{i}}=\frac{\partial U^{T}}{\partial \alpha_{i}}KU+U^{T}\frac{\partial K}{\partial \alpha_{i}}U+U^{T}K\frac{\partial U}{\partial \alpha_{i}}$$

Taking derivatives on Equation (5), we obtain:

(8) $$\frac{\partial U}{\partial \alpha_{i}}=-K^{-1}\frac{\partial K}{\partial \alpha_{i}}U$$

Then, putting Equation (8) into Equation (7), we have:

(9) $$\frac{\partial c\left(\gamma ,\alpha \right)}{\partial \alpha_{i}}=-U^{T}\frac{\partial K}{\partial \alpha_{i}}U$$

The derivative of stiffness matrix K on $\alpha_{i}$ can be calculated with the chain rule, as:

(10) $$\frac{\partial K}{\partial \alpha_{i}}=\frac{\partial K}{\partial \rho_{j}}\frac{\partial \rho_{j}}{\partial \alpha_{i}}$$

Referring to Equations (3) and (4), we have:

(11) $$\frac{\partial K}{\partial \rho_{j}}=\frac{\gamma_{i}^{p_{1}}\left(1+p\right)}{{\left[1+p_{2}(1-\rho_{j})\right]}^{2}}(E_{0}-E_{min})K_{0}$$

The derivative of $\rho_{j}$ on $\alpha_{i}$ can be expressed through:

(12) $$\frac{\partial \rho_{j}}{\partial \alpha_{i}}=\frac{\partial \rho_{j}^{off}}{\partial \alpha_{i}}-\frac{\partial \rho_{j}^{ned}}{\partial \alpha_{i}}$$

in which the derivatives of $\rho_{j}^{off}$ and $\rho_{j}^{ned}$ can be derived by referring to Equation (1). Finally, combining Equations (9)–(12) reaches the following result:

(13) $$\frac{\partial c\left(\gamma ,\alpha \right)}{\partial \alpha_{i}}=-U^{T}\left[\frac{\gamma_{i}^{p_{1}}\left(1+p\right)}{{\left[1+p\left(1-\rho_{j}\right)\right]}^{2}}\left(E_{0}-E_{min}\right)K_{0}\right]U\left(\frac{\partial \rho_{j}^{off}}{\partial \alpha_{i}}-\frac{\partial \rho_{j}^{ned}}{\partial \alpha_{i}}\right)$$

Note that the smoothing filter is applied to α to prevent its sharp changes, and in this research, the smoothing filter also functions to control the maximum drawing angle. Hence, the following filter function [1] is adopted:

(14) $$\tilde{\alpha_{e}}=\frac{1}{\sum_{k\in N_{e}}H_{ek}}\sum_{k\in N_{e}}H_{ek}\alpha_{k}\;H_{ek}=max\left(0,\;r-∆(e,k)\right)$$

where $N_{e}$ means the set of elements that fall inside a circular area centered at the element e with the radius r. ∆(e,k) means the distance between the elements e and k.

Then, the sensitivity result will be adjusted to:

(15) $$\frac{\partial c\left(\gamma ,\alpha \right)}{\partial \alpha_{i}}=\sum_{e\in N_{i}}\frac{\partial c\left(\gamma ,\alpha \right)}{\partial \tilde{\alpha_{e}}}\;\frac{\partial \tilde{\alpha_{e}}}{\partial \alpha_{i}}=\sum_{e\in N_{i}}\frac{1}{\sum_{k\in N_{e}}H_{ek}}H_{ei}\frac{\partial c\left(\gamma ,\alpha \right)}{\partial \tilde{\alpha_{e}}}$$

Similarly, the sensitivity of the objective function on $\gamma_{i}$ is obtained following the procedures. The smoothing filter will be applied to γ as well, but the details are omitted here for the sake of brevity.

(16) $$\frac{\partial c\left(\gamma ,\alpha \right)}{\partial \gamma_{i}}=-U^{T}p_{1}\gamma_{i}^{p_{1}-1}\frac{\rho_{i}}{1+p_{2}\left(1-\rho_{i}\right)}\left(E_{0}-E_{min}\right)K_{0}U$$

With the sensitivity results, the method of moving asymptotes (MMA) is applied to update the truncation variables α, and the step length equals the density difference (measured based on $\rho_{tmp}$) of two neighboring elements in a column. The optimality criteria (OC) method [1,50] is adopted to update γ with the step length of 0.2.

### 2.4. Uniform Thickness Control

The uniform thickness is a general feature of stamping structures, i.e., the thickness along the surface normal direction should stay constant. From the above, it can be seen that ∆α determines the thickness along the stamping direction; however, the stamping direction is not always aligned with the surface normal, indicating that the uniform ∆α may not lead to a constant thickness. Hence, we propose to offset the surface that is derived by truncating with α by a constant distance along the surface normal to obtain the varying ∆α for uniform thickness control.

**Figure 4:**
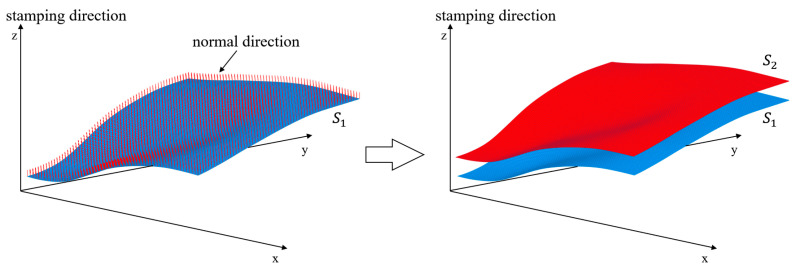
Schematic plot of the surface offset with MATLAB R2020b (https://www.mathworks.com/products/matlab.html).

Specifically, the truncation based on α creates a surface $S_{1}$ (e.g., the blue-colored surface in Figure 4) by identifying the truncation z-coordinate for each column and performing the polynomial fitting on the column-wise truncation points. Then, with the polynomial function, the surface normal can be analytically calculated and the truncation points will be deviated along the surface normals through a fixed distance d, the target thickness, to generate the set of sample points belonging to the offset surface $S_{2}$. The polynomial fitting through regression analysis will be performed again to express the offset surface (e.g., the red-colored surface in Figure 4). In this way, the varying ∆α can be obtained through:

(17) $$∆\alpha =\frac{S_{2}-S_{1}}{∆z}$$

where ∆z is the design domain thickness.

## 3. Numerical Examples

### 3.1. Case 1

**Figure 5:**
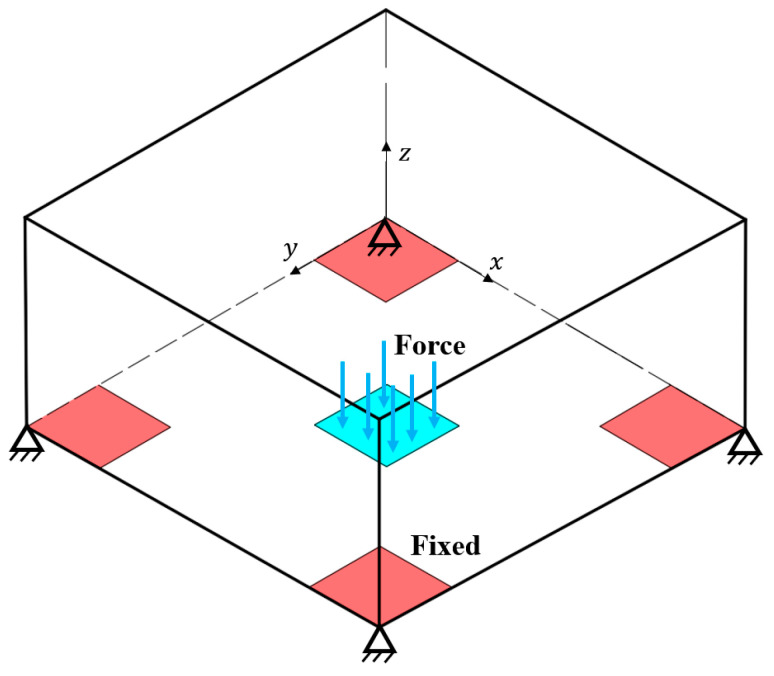
The design domain (120 mm × 120 mm × 20 mm) and boundary conditions.

The design domain and boundary conditions of the first case are illustrated in Figure 5. The four foot corners of the bottom surface are fixed. A force F = 4 kN is uniformly distributed in the center area of the bottom surface. The design domain of 120 mm × 120 mm × 20 mm is discretized with 120 × 120 × 20 bilinear hexahedral elements. The base material has the property parameters of Young’s modulus $E_{0}$ = 1 GPa and Poisson ratio μ = 0.3. In the optimization setup, the target part thickness is set to 4 mm, and the maximum volume fraction is 0.1. The smoothing filter radii for γ and α are set to 4 and 6 times the element size, respectively. The β in the Heaviside projection increases throughout the iterations, starting from 1, doubling every 40 iterations or when the objective function changes less than 0.1%, and ending at the threshold 64. The update step length for α is 0.05, and the optimization terminates when the objective function varies less than 0.1% in the consecutive iterations. Note that all algorithms are programmed and implemented in Matlab.

**Figure 6:**
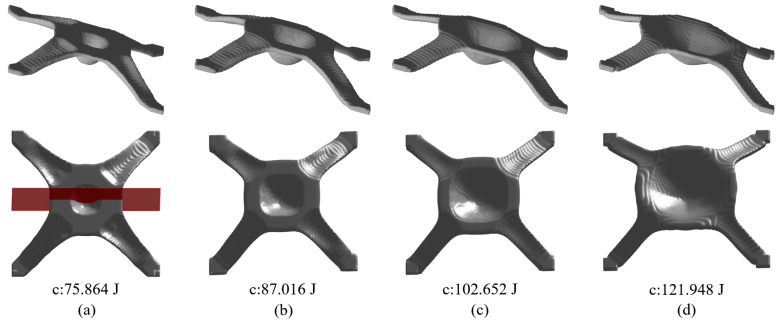
Optimization results and their corresponding objective function values with different smoothing radii r for α: (**a**) r=6, (**b**) r=10, (**c**) r=14, (**d**) r=18.

**Figure 7:**
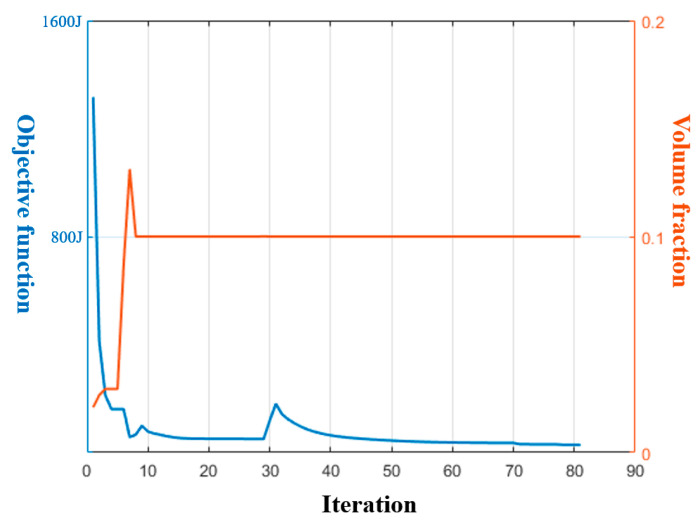
Convergence history for Figure 6a.

**Figure 8:**
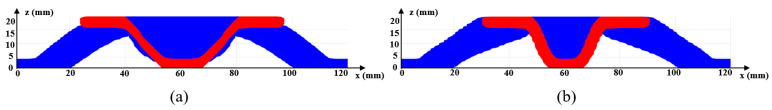
Cross section views for Figure 6a to show the thickness control effect with (**a**) a consistent ∆α and (**b**) the varying ∆α from surface offset.

Figure 6a gives the optimized material distribution and Figure 7 shows the associated convergence history. The final structural compliance is 75.864 J. As observed from the convergence curves, the objective function keeps the reducing trend for the majority of iterations while at the iterations in which β (for Heaviside projection) updates, the objective value jumps (around iteration 30) or steps down (around iteration 70). This converging phenomenon is reasonable since we cannot set a large β from the beginning because of the associated convergence instability, while a small β would lead to a final blurred boundary. To evaluate the thickness control effect, a cross section of the result in Figure 6a is demonstrated in Figure 8b, in which the red color means the cross section and the blue color represents the solids behind the cross section. Apparently, the uniform thickness effect is well achieved with the surface fitting and offset strategy to dynamically determine the ∆α. In contrast, the design effect with a constant ∆α is demonstrated in Figure 8a. The thicknesses of the platen areas are consistent but the thicknesses for areas with a drawing angle would be thinned, thus deviating from the uniform thickness requirement.

**Figure 9:**
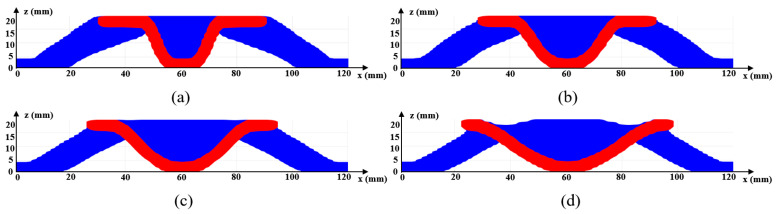
Cross section views to show the drawing angle control effect with (**a**) r=6, (**b**) r=10, (**c**) r=14, (**d**) r=18.

Regarding the proposed method, an important parameter to investigate is the filter radius for smoothing α; referring to Equation (14). The parameter α is smoothed to prevent its sudden changes, thus avoiding sharp peaks or valleys. In general, an intermediate value is suitable for α, since a small radius does not smear out the sharp changes and a large radius prevents material accumulation at the top and bottom surfaces. In this case, the range of 6~20 is assigned to the filter radius. The optimization results subject to the varying filter radii are presented in Figure 6. Apparently, a larger filter radius leads to increased structural compliance due to the reduced space for distributing α. More importantly, the filter radius has an impact on the maximum drawing angle, i.e., referring to the cross section views in Figure 9, a larger filter radius leads to a smaller drawing angle and even though the structural compliance is increased, the manufacturability has been improved owing to fewer plastic deformations.

**Figure 10:**
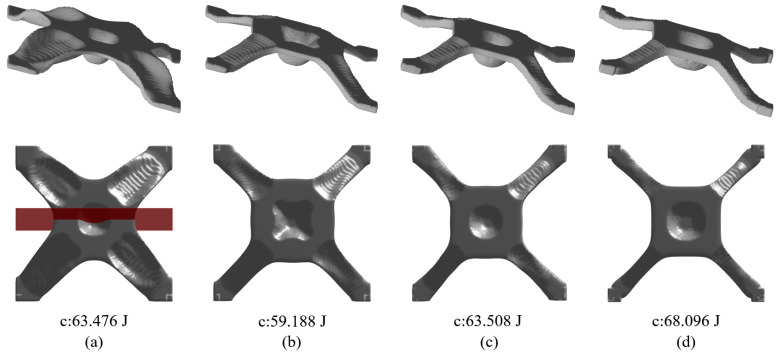
Optimization results and their corresponding objective function values subject to different thickness targets of (**a**) Th = 4 mm, (**b**) Th = 5 mm, (**c**) Th = 6 mm, and (**d**) Th = 7 mm.

**Figure 11:**
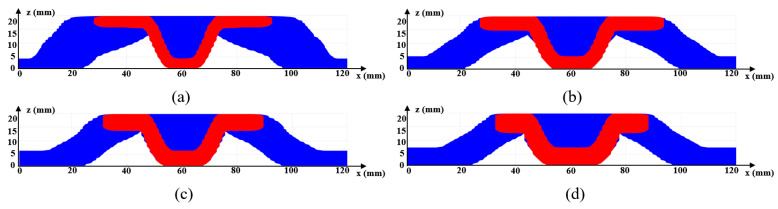
Cross section views for Figure 10 to show the controlled uniform thickness with (**a**) Th = 4 mm, (**b**) Th = 5 mm, (**c**) Th = 6 mm, and (**d**) Th = 7 mm.

At the end, we perform one more set of numerical trials with different thickness targets, i.e., Th = 4 mm, 5 mm, 6 mm, and 7 mm. To ensure the proper convergence by avoiding tiny features, the volume fraction upper limit is increased to 0.15. The optimization results are presented in Figure 10, and the cross section views are demonstrated in Figure 11. As can be identified, varying the target thickness leads to major differences among the optimization results, indicated by the associated structural compliances, and Th = 5 mm is the best amongst the tests that has the optimized structural compliance of 59.188 J. This raises an open question about simultaneously optimizing the shell topology and thickness that can lead to a better optimization effect and is worthy of future investigation. Nevertheless, the thickness control effect is perfectly achieved for all scenarios.

### 3.2. Case 2

**Figure 12:**
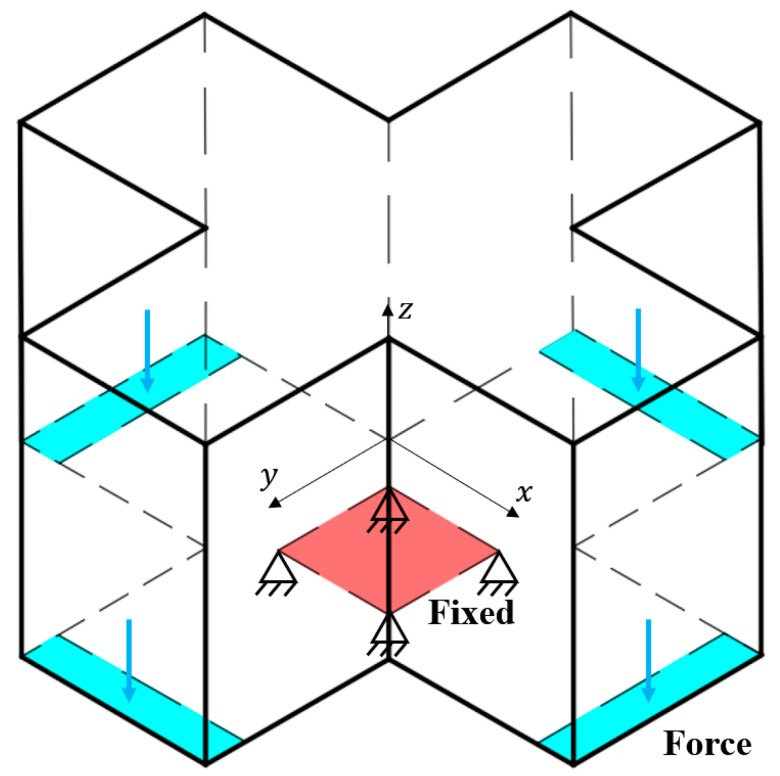
The design domain (has the size of 120 mm × 120 mm × 20 mm) and boundary conditions of case 2.

Figure 12 introduces the design domain and boundary conditions of the second case. The squared center area of the bottom surface is fixed for zero displacement. A total force F = 4 kN is uniformly loaded onto the four side edges of the bottom surface. The design domain of 120 mm × 120 mm × 20 mm is discretized with 120 × 120 × 20 bilinear hexahedral elements. Young’s modulus $E_{0}$ and Poisson ratio μ of the base material are set to 1 GPa and 0.3, respectively. Given the optimization setup, the target part thickness is still Th = 4 mm, and the volume fraction upper limit is set to 0.15. The smoothing filter radii for γ and α are set to 4 and 8 times the element size, respectively. The β in Heaviside projection increases throughout the iterations, starting from 1 and doubling every 40 iterations or when the objective function changes less than 0.1% until reaching the threshold 64. The update step length for α is 0.05, and the optimization terminates when the objective function varies less than 0.1% in consecutive iterations.

**Figure 13:**
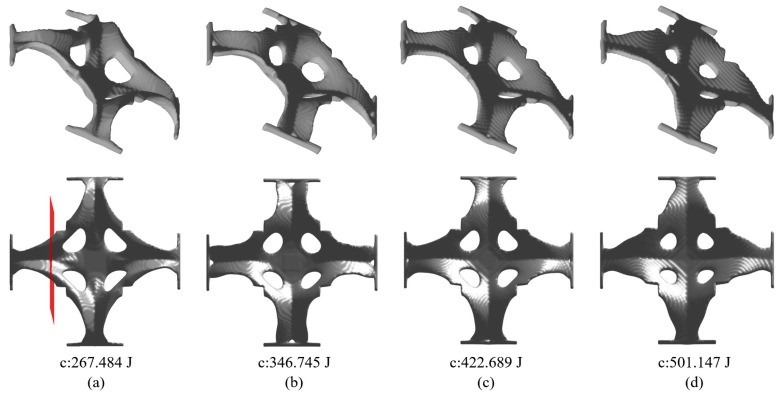
Optimization results and their corresponding objective function values with different smoothing radii r for α: (**a**) r=8, (**b**) r=12, (**c**) r=16, (**d**) r=20.

**Figure 14:**
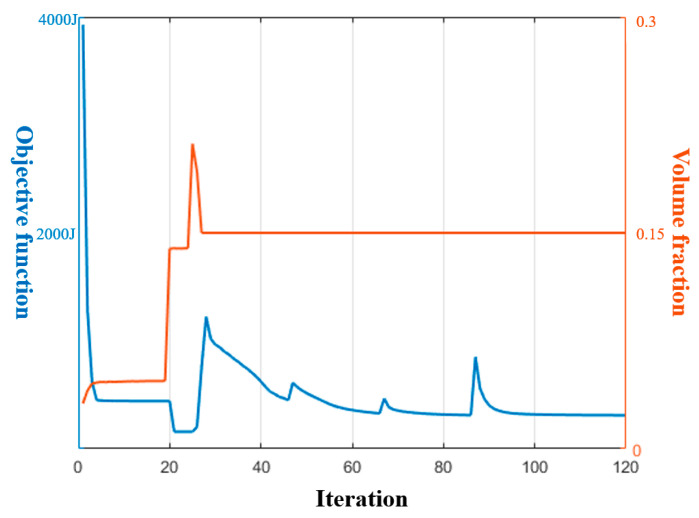
Convergence history for Figure 13a.

**Figure 15:**
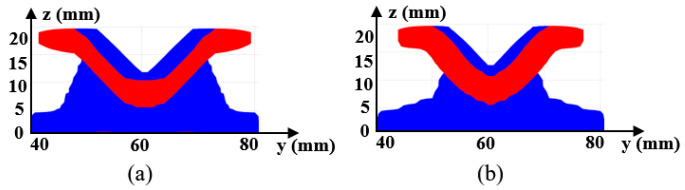
Cross section views for Figure 13a to show the thickness control effect with (**a**) a consistent ∆α and (**b**) the varying ∆α from surface offset.

The optimized shell structure is demonstrated in Figure 13a. Figure 14 shows the convergence history with the final compliance of 267.484 J. The structural pattern is effective in bearing load since four connecting bars are formed as the load transfer path and four other struts connect the bars to enhance the deformation resistance. The convergence curves show a consistently reduced objective value, indicating the effectiveness in searching for an optimum solution. The cross section views of the optimized structure for Figure 13a subject to different thickness control methods are given in Figure 15. The red color means the cross section and the blue color represents the solids behind the cross section. Similar conclusions can be drawn that imposing a consistent ∆α fails to achieve uniform thickness control.

**Figure 16:**
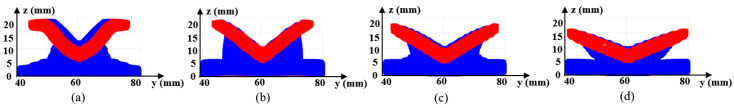
Cross section views to show the drawing angle control effect with (**a**) r=8, (**b**) r=12, (**c**) r=16, (**d**) r=20.

Figure 13 also demonstrates the optimization results subject to the different smoothing filter radii for α. The resulting structural compliance increases rapidly with the enlarged radius due to the severely reduced design space. A larger smoothing radius for α leads to less variation of the stamping depths and, therefore, the connecting bars would have a shallower V-shaped cross section (see Figure 16), for which the bending deformation resistance is weakened. On the other hand, the smoothing radius affects the maximum drawing angle of the obtained structure, since a larger filter radius leads to more column design variables participating in the drawing depth averaging, resulting in a smaller drawing angle (see Figure 16) and, therefore, improved manufacturability.

**Figure 17:**
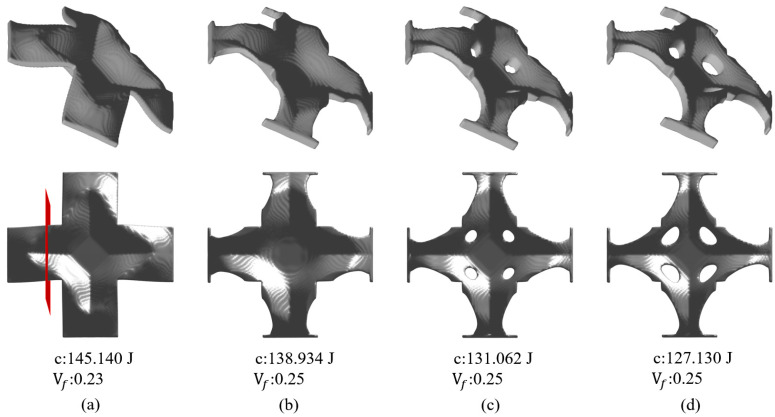
Optimization results and their corresponding objective function values subject to different thickness targets of (**a**) Th = 4, (**b**) Th = 5, (**c**) Th = 6, and (**d**) Th = 7.

**Figure 18:**
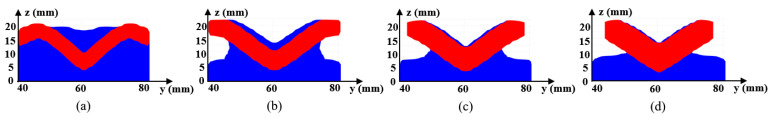
Cross section views for Figure 17 to show the controlled uniform thickness with (**a**) Th = 4 mm, (**b**) Th = 5 mm, (**c**) Th = 6 mm, and (**d**) Th = 7 mm.

At the end, the trials with varying target thicknesses are performed. The set of target thicknesses of Th = 4 mm, 5 mm, 6 mm, and 7 mm is considered. The volume fraction upper limit is increased to 0.25. Accordingly, the optimization results are shown in Figure 17, and the cross section views are shown in Figure 18. The result of Figure 17a does not meet the material usage upper limit since the domain is already fully filled. For the other three, the allowable materials are fully used and a higher wall thickness leads to more topological variable evolution to satisfy the maximum volume fraction constraint. For instance, materials were removed to change the boundary shape of the stamped panel, as shown in Figure 17b, and topological changes happen to the panel when Th increased beyond 6, as shown in Figure 17c,d, when four open holes were made in the panel. Overall, the increased target thickness plus topological changes lead to reduced structural compliance. The uniform wall thickness is perfectly achieved in all scenarios.

The above results show that the proposed method can perform topology optimization of stamping structures with tunable uniform thickness and maximum drawing angle control. No internal voids or undercuts appear in the results.

## 4. Conclusions

This paper presents a shape and topology optimization method for stamping structures. The novel directional density field plus two consecutive truncations are proposed to define the physical density field that facilitates directional material removal and uniform thickness control. The maximum drawing angle is proved tunable by adjusting the filter radius of the truncation threshold, indicating the adjustable balance between performance and manufacturability. The effectiveness of the established method has been proved by a few numerical case verifications. The main conclusion points are summarized below:

(1) The directional density field, realized by the multiple Heaviside projections, is effective in enabling directional material adding and removal. This type of material change ensures no interior void or undercut and, therefore, is very suited for designing stamping structures.
(2) The polynomial-fitting-based surface offset provides the dynamic truncation threshold offsets that have proved more effective in uniform thickness control than keeping a constant truncation threshold offset because the surface normal vectors are not always aligned with the stamping direction.
(3) The smoothing radius for the truncation threshold majorly affects the optimization result. A larger smoothing radius for α leads to less variation in stamping depth and, therefore, a shallower V-shaped cross section, for which the bending deformation resistance is weakened. On the other hand, a larger radius leads to more column design variables participating the drawing depth averaging, resulting in a smaller drawing angle and, therefore, improved manufacturability due to the fewer plastic deformations.
(4) Varying the target thickness of the thin-walled structure leads to major changes in the optimization result, but there is no deterministic rule for pre-defining the best target thickness, which varies case by case. Hence, simultaneously optimizing the shell topology and thickness would be a very interesting future topic.
(5) The proposed topology optimization algorithm exhibits excellent convergence stability. Minor fluctuations have been observed which are reasonable given the continuously increasing β strategy.

In future work, the cracking during stamping due to material incoherency should be considered to facilitate the design–manufacture consistency. Incorporating an elastoplastic constitutive model, addressing geometric and material nonlinearity, and adding ultimate strength constraints would be the critical techniques. Additionally, buckling strength should also be involved due to the frequent buckling failures of thin-walled stamping structures.

## Data Availability

The code will be partially available on request.
